# High-Resolution, High-Throughput, Positive-Tone Patterning of Poly(ethylene glycol) by Helium Beam Exposure through Stencil Masks

**DOI:** 10.1371/journal.pone.0056835

**Published:** 2013-05-24

**Authors:** Eliedonna E. Cacao, Azeem Nasrullah, Tim Sherlock, Steven Kemper, Katerina Kourentzi, Paul Ruchhoeft, Gila E. Stein, Richard C. Willson

**Affiliations:** 1 Department of Chemical and Biomolecular Engineering, University of Houston, Houston, Texas, United States of America; 2 Department of Electrical and Computer Engineering, University of Houston, Houston, Texas, United States of America; 3 Department of Biomedical Engineering, University of Houston, Houston, Texas, United States of America; 4 Department of Biology and Biochemistry, University of Houston, Houston, Texas, United States of America; 5 The Methodist Hospital Research Institute, Houston, Texas, United States of America; University of California, Merced, United States of America

## Abstract

In this work, a collimated helium beam was used to activate a thiol-poly(ethylene glycol) (SH-PEG) monolayer on gold to selectively capture proteins in the exposed regions. Protein patterns were formed at high throughput by exposing a stencil mask placed in proximity to the PEG-coated surface to a broad beam of helium particles, followed by incubation in a protein solution. Attenuated Total Reflectance–Fourier Transform Infrared Spectroscopy (ATR–FTIR) spectra showed that SH-PEG molecules remain attached to gold after exposure to beam doses of 1.5–60 µC/cm^2^ and incubation in PBS buffer for one hour, as evidenced by the presence of characteristic ether and methoxy peaks at 1120 cm^−1^ and 2870 cm^−1^, respectively. X-ray Photoelectron Spectroscopy (XPS) spectra showed that increasing beam doses destroy ether (C–O) bonds in PEG molecules as evidenced by the decrease in carbon C1s peak at 286.6 eV and increased alkyl (C–C) signal at 284.6 eV. XPS spectra also demonstrated protein capture on beam-exposed PEG regions through the appearance of a nitrogen N1s peak at 400 eV and carbon C1s peak at 288 eV binding energies, while the unexposed PEG areas remained protein-free. The characteristic activities of avidin and horseradish peroxidase were preserved after attachment on beam-exposed regions. Protein patterns created using a 35 µm mesh mask were visualized by localized formation of insoluble diformazan precipitates by alkaline phosphatase conversion of its substrate bromochloroindoyl phosphate-nitroblue tetrazolium (BCIP-NBT) and by avidin binding of biotinylated antibodies conjugated on 100 nm gold nanoparticles (AuNP). Patterns created using a mask with smaller 300 nm openings were detected by specific binding of 40 nm AuNP probes and by localized HRP-mediated deposition of silver nanoparticles. Corresponding BSA-passivated negative controls showed very few bound AuNP probes and little to no enzymatic formation of diformazan precipitates or silver nanoparticles.

## Introduction

Surface patterning of biomolecules is important in the study of cell adhesion, in tissue engineering, and in the development of diagnostics and biomedical assays such as protein nanoarrays [1], [2], [3], [4]. Controlled attachment of biomolecules can be achieved by approaches generally categorized as “top-down” (e.g., photolithography, focused ion beam lithography, electron beam lithography, nanografting), or “bottom-up” (e.g., self-assembly of monolayers, dip-pen nanolithography, micro/nano contact printing or stamping), or combinations of these techniques. Top-down techniques manipulate an instrument to modify the bulk material to create patterns. Photolithography is a high-throughput lithographic process but its resolution is diffraction-limited below the micron-scale, and it is expensive to use for low-volume manufacturing. Electron beam and focused ion beam lithography have very high resolution but very low throughput. Bottom-up techniques take advantage of the spontaneous organization of molecules to produce a more complex and functional patterned material. These methods can allow patterning below the current resolution of lithographic techniques, but often involve high costs (for large areas) and imperfect patterning. Current approaches to biomolecule patterning, therefore, often employ combinations of top-down and bottom-up techniques [4], [5], [6].

Several approaches have been taken to biomolecule, specifically protein, patterning on surfaces. Utilizing self-assembly and electron-beam lithography, Geyer et al. [7] and Golzhauser et al. [8] have demonstrated the use of nitro-terminated aromatic thiols assembled on gold surfaces to create surface patterns of chemical functionality. Upon exposure to the electron beam, the nitro groups are converted to amines while the underlying aromatic groups are dehydrogenated and cross-linked. The amine groups were then used to attach molecules (carboxylic acid anhydrides and rhodamine dyes) to the exposed regions of the surface. Perhaps the most popular approach, however, involves the use of poly(ethylene glycol) (PEG) [9], [10], [11], which is well-known for its resistance to non-specific adsorption of proteins through a combination of entropic (steric stabilization) and enthalpic (hydrogen bonding) mechanisms [12], [13]. Entropic passivation is favored by longer chains which can assume a greater variety of configurations [13], [14], [15], but even smaller ethylene oxide chains of 3–6 monomer units can resist protein adsorption as long as the molecular conformation is helical or amorphous, favoring a more stable interfacial layer of tightly-bound water [12], [13], [15], [16], [17], [18].

Electron beam lithography is commonly used to create protein patterns on PEG surfaces. Hong et al. [19] observed that proteins selectively attach to electron beam-exposed regions of amine-terminated PEG spin-coated on silicon. Patterns consisting of multiple proteins also can be formed by introducing PEG bearing different functionalities (e.g., biotin, maleimide, aminooxy or metal chelate) that are patterned over several exposures [20]. Harnett et al. [21] used low-energy electron beam exposure to destroy the amine functionality in selected regions of an amine-functionalized silane-PEG on silicon surfaces, allowing proteins to attach only on the unexposed regions (negative-tone patterning). Inert silane-PEG on silicon and thiol-PEG on gold have also been used to create a protein-resistant surface, with electron beam exposure used to remove the SAM on exposed regions and protein-reactive PEGs used to backfill the exposed regions (positive-tone patterning) [22], [23].

In this study, we explored the use of helium beam proximity lithography to create micron and nanoscale patterns of proteins on grafted PEG in a high-throughput manner. Unlike focused ion beam lithography techniques, proximity printing forms its patterns by exposing a thin membrane with etched openings corresponding to the desired pattern (a “stencil mask”) to a broad beam of helium particles, which are either stopped in the opaque regions of the mask or pass through the openings. In this way, the pattern can be formed very quickly and without the need for expensive ion optics. After exposure to the beam, protein patterns were detected and visualized using three different methods chosen to be representative of common approaches: (1) formation of localized diformazan precipitates by patterned alkaline phosphatase (AP) from its substrate BCIP-NBT [24], [25], [26], (2) binding of gold nanoparticle probes (40 nm and 100 nm gold particles conjugated with antibodies) [27], [28], [29], by patterned antibodies and avidin, and (3) localized redox formation of silver deposits mediated by patterned horseradish peroxidase (HRP) [30], [31], [32], [33].

## Materials and Methods

### Materials

Alkaline phosphatase from bovine intestinal mucosa (Product number P6772), goat polyclonal anti-rabbit antibodies (R2004), goat polyclonal anti-mouse antibodies (M8645), hen egg white lysozyme (L6876) and BCIP-NBT phosphatase substrate (72091) were purchased from Sigma-Aldrich (St. Louis, MO), while avidin (21121) was from Pierce Protein Research Products, Thermo Scientific (Rockford, IL). Rabbit polyclonal anti-*Rickettsia* antibodies were generously supplied by Dr. Juan Olano (University of Texas Medical Branch, Galveston, TX) and mouse anti-lysozyme monoclonal D1.3 antibody [34] was produced for us in hollow-fiber hybridoma culture by Biovest (Minneapolis, MN). Streptavidin-horseradish peroxidase (strep-polyHRP80, 65R-S105PHRP) conjugate from Fitzgerald Industries International (Action, MA) is a polymer consisting of 80 streptavidin molecules per conjugate and 5 HRP monomer molecules per streptavidin giving an estimated total of 400 HRP molecules per conjugate. Biotinylated antibodies and AP were prepared using the EZ-Link Sulfo-NHS-LC-Biotinylation Kit (21435, Pierce Protein Research Products, Thermo Scientific, Rockford, IL). Gold nanoparticle probes were prepared via EDC-NHS chemistry (based on the manufacturer’s protocol) on carboxylated 100 nm gold nanoparticles (20-PC-100, Nanopartz Inc., Loveland, CO). Thiol-polyethylene glycol (MW 5000, ∼112 PEG monomers, PEG3-0021) was from Nanocs (Boston, MA). HRP redox silver staining solution (6010, EnzMet^TM^ staining kit) was purchased from Nanoprobes, Inc. (Yaphank, NY).

### Preparation of assembled thiol-polyethylene glycol on gold

Gold surfaces were prepared by thermally evaporating 100 nm gold (with a 20 nm nickel-chromium adhesion layer) on 4-inch silicon wafers. Before any further treatment, these gold surfaces were cleaved (*ca.* 4 cm^2^) and cleaned by dipping into anhydrous ethanol for at least 2 minutes, then thoroughly rinsed with deionized water (18 MΩ), and finally dried with a stream of compressed nitrogen. The clean surfaces were then immersed in a 1 mM solution of SH-PEG in 90% ethanol and allowed to incubate overnight (at least 18 hours) at room temperature. Afterwards, the surfaces were washed thrice with DI water and dried with compressed nitrogen.

### Helium beam exposure of PEG and surface characterization

The helium beam is generated in a saddle-field ion source that is based on the designs of Hogg [35] and Franks [36]. In this source, a plasma is ignited at low pressure when electrons are trapped along long oscillatory paths through a saddle-point in the electric potential distribution, and ions escape through a small aperture machined into one end of the source. As the ions escape, a fraction is neutralized through charge-exchange collisions with the neutral helium gas ambient and a beam of mixed atoms and ions drifts along the length of a vacuum beam line to an exposure chamber, located about 1.5 meters from the source [37]. To form patterns, a stencil mask is held in proximity to the wafer by a fixture and the wafer can be moved below the mask using an in-vacuum x-y stage to allow for step-and-repeat patterning of large surfaces. The exposure time is controlled through a computer that actuates a mechanical shutter.

**Figure 1:**
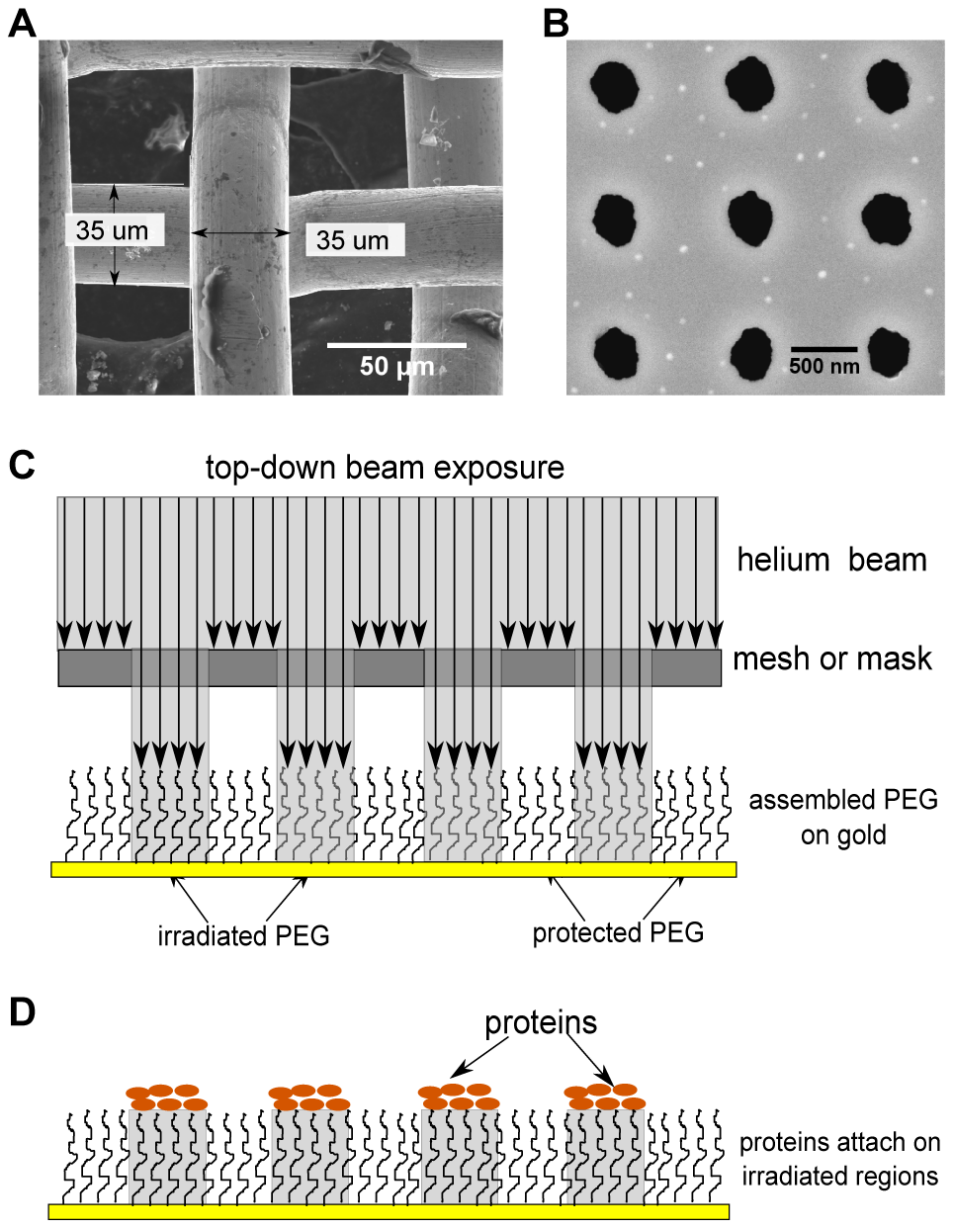
A schematic of helium beam-induced protein patterning on PEG. Panels A and B show the electron images of 35 µm mesh and 300 nm masks, respectively. Top-down helium beam exposure through a mask (C) of grafted PEG on surface allowed proteins preferentially to attach on irradiated regions (D) to form patterns.

Patterns of beam-modified PEG (and, subsequently, proteins) on the gold surfaces were formed by casting shadows using a stencil mask in proximity to the substrate (see Figure 1). The mixed ion and atom flux was equivalent to a helium ion beam current density of about 70 nA/cm^2^ with a beam energy of about 6.5 keV (for a source power supply setting of 10 kV at 1 mA), and three doses were tested: 30, 150 and 600 seconds ( approximately 2, 10 and 45 µC/cm^2^, respectively). After exposure, samples were stored dry at 4°C until use. Surfaces were analyzed by Attenuated Total Reflectance-Fourier Transform Infrared Spectroscopy, ATR-FTIR (Nicolet 4700 FT-IR, Thermo Scientific) before and after beam exposure, and after PBS buffer incubation. In addition, surfaces before and after incubation with protein (15 µg/mL avidin) were analyzed by X-ray Photoelectron Spectroscopy (Physical Electronics model 5700 XPS instrument) using a monochromatic Al-kα X-ray source (1486.6 eV) operated at 350 W. The analyzed area, collection solid cone and take-off angle were set at 0.8 mm diameter, 5° and 45°, respectively. Applying a low pass energy filter of 11.75 eV resulted in an energy resolution of better than 0.51 eV. All spectra were acquired at room temperature under a vacuum of 5×10^−9^ torr or better. A survey scan was first performed to determine the major elements present, followed by element-specific scans of at least 15 minutes per scan. Data processing was carried out using the Multipak^TM^ software package (Physical Electronics, Inc.). A Shirley background [38] subtraction routine was applied.

### Protein patterning on PEG

After helium beam exposure, surfaces were further cleaved into smaller pieces (*ca.* 25 mm^2^) to fit into micro-centrifuge tubes. Samples used for patterning were incubated with 15 µg/mL of protein solution (either avidin, streptavidin-polyHRP, goat anti-mouse antibodies or goat anti-rabbit antibodies) in PBS buffer for 1 hour at room temperature, while negative control surfaces were incubated with protein-free PBS buffer. Before pattern detection, all surfaces were immersed in 4% BSA in PBS for 1 hour to further passivate the back and edges of the gold-coated silicon chips, as well as the walls of the micro-centrifuge tube. All surfaces were washed at least three times with PBS (except where specified) between reagents.

For pattern detection by formation of alkaline phosphatase diformazan precipitate, surfaces were incubated on an orbital shaker in a solution of 2 µg/mL of biotinylated enzyme (with *ca.* 3 biotin molecules per enzyme molecule, as assessed by HABA assay [39]) in 100 mM diethanolamine, 100 mM NaCl, 5 mM MgCl_2_, pH 9.2 (DEA buffer), for 1 hour at room temperature, then washed with DEA buffer. BCIP-NBT (0.15 mg/mL BCIP and 0.30 mg/mL NBT) substrate was then added and allowed to react for 10 minutes. The surfaces were then washed with water, dried with compressed nitrogen, and imaged using an optical microscope. For pattern testing by gold nanoparticle probes, 300 µL of a suspension of *ca.* 10^9^ gold nanoparticles/mL conjugated with antibodies (100 nm particles with biotinylated goat anti-rabbit polyclonal antibodies, or 40 nm particles with goat anti-mouse antibodies) in PBS buffer was added to the surface and allowed to incubate for at least 12 hours at room temperature, with mixing on an orbital shaker. For HRP-mediated silver staining, surfaces were incubated with streptavidin-polyHRP conjugates (20 µL spot, 10 µg/mL) for at least one hour at room temperature, and then washed thrice with PBS and twice with DI water. Surfaces were then silver-stained using the EnzMet^TM^ staining solution, with 2 minutes incubation time each for the manufacturer’s reagents Detect A, B and C (20 µL each). Finally, surfaces were washed thrice with water, dried with compressed nitrogen and imaged by scanning electron microscopy (Zeiss/LEO 1525 Field Emission SEM).

## Results and Discussion

**Figure 2:**
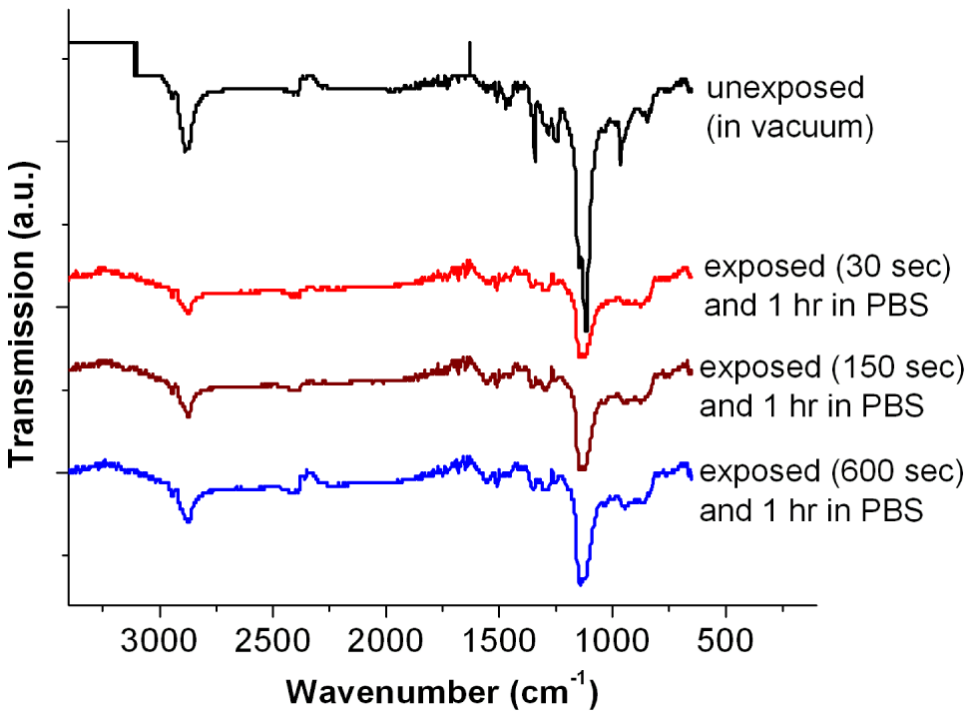
ATR-FTIR spectra of beam protected (unexposed) and irradiated (exposed) PEG at different helium beam doses. Spectra of grafted PEG subjected to different beam doses and incubated in PBS for 1 hour show the characteristic ether and methoxy peaks at 1120 cm^−1^ and 2870 cm^−1^, respectively, which also are present in unexposed PEG.

As shown in Figure 2, ATR spectra confirmed the presence of SH-PEG assembled on gold surfaces before helium beam exposure, after beam exposure and after post-exposure incubation in PBS buffer for one hour. Peaks near 2870 cm^−1^ and 1120 cm^−1^ were observed, corresponding to the C–H stretch of methoxy and C–O stretch of ether groups, respectively [40]. We found that our MW 5000 SH-PEG SAM monolayers at least partially survived 6.5 kV helium beam exposures with doses of 1.5–60 µC/cm^2^. It is notable that previous researchers [22], [23] found that monolayers of smaller PEG molecules (MW: 290 and 750) were damaged by doses of 5–80 µC/cm^2^ and completely removed by doses over 160 µC/cm^2^ when using 1 kV electrons.

**Figure 3:**
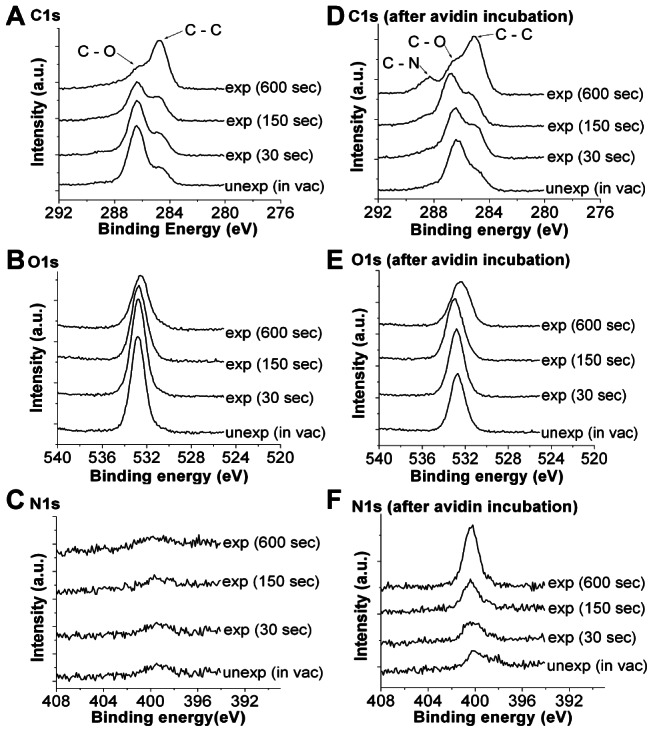
Elemental XPS spectra of beam protected (unexposed) and irradiated (exposed at different doses) PEG before and after protein incubation. Before (“unexp”) and after (“exp”) helium beam exposure, carbon C1s signals (A) show characteristic alkyl and ether peaks at 284.6 eV and 286.6 eV binding energies, respectively. The presence of oxygen O1s signals (B) at 532 eV and the absence of nitrogen N1s signals (C) at 400 eV also were observed. Subsequent incubation with avidin shows additional C1s peak at 288 eV (D), similar O1s signals at 532 eV (E) and existence of N1s peak at 400 eV (F) binding energies for beam exposed PEG.

The radiation chemistry of electron and ion/atom beams on PEG and alkanethiols would be expected to be very similar, mainly involving hydrogen abstraction of the PEG hydrocarbons [41] and dissociation of C–H, C–C, C–S and substrate–SH bonds [23], [34]. Hydrogen abstraction and bond dissociation form radicals, which eventually cause cross-linking within the polymer [42], [43], affect hydrogen bonding and disrupt the highly organized interface between water and PEG. Our results are consistent with a similar effect of helium beam exposure. Cross-linking of PEG was observed on helium beam exposed surfaces as evidenced by the water insolubility of exposed spin-coated thiol-PEG on silicon (unexposed thiol-PEG film on silicon is soluble). Furthermore, disruption of PEG chains was confirmed by XPS, as shown in Figure 3. As presented in Figure 3A, prominent carbon (C1s) peaks were observed at 284.6 eV and 286.6 eV binding energies, which correspond to alkyl and ether C1s bonding states, respectively [44], [45]. As the helium beam dose was increased, the C1s ether peak at 286.6 eV decreased while the alkyl peak at 284.6 eV increased, indicating the destruction of ether bonds and formation of more alkyl bonds in the PEG molecules. A decrease in the oxygen (O1s) signal (532 eV binding energy) also was observed as the beam dose was increased. Helium beam exposure renders the grafted PEG on the surface less hydrophilic by a decrease in C–O ether and an increase in C–C alkyl functionalities, and thus potentially more amenable to protein adsorption. There also is literature evidence that electron [9] or ion (argon) beam [10], [11] exposure can create carbonyl functionalities (which could be charged carboxylate or protein amine-aldehyde) in PEG samples. A relatively small number of these functional groups could alter the local protein-capture properties of the PRG surface, while being difficult to detect against the background of much-more-numerous ether and alkyl functionalities.

Elemental XPS spectra of exposed and unexposed samples incubated with avidin are shown in Figures 3D, 3E and 3F for carbon (C1s), oxygen (O1s) and nitrogen (N1s) signals, respectively. In Figure 3D, aside from the observed decrease in the C1s peak at 286.6 eV and increase in the C1s peak at 284.6 eV discussed previously, the C1s peak at 288 eV, a characteristic binding energy of amide C1s [46], appeared as the helium beam dose was increased, confirming that avidin attaches to the beam-exposed surfaces. This is further supported by the increase in the nitrogen N1s signal (400 eV) with increasing beam dose, as shown in Figure 3F. Unlike in Figure 3B, the differences in oxygen signal intensities in Figure 3E were not distinct, particularly in the unexposed and exposed (30 sec and 150 sec) samples. This might be due to the added attenuation length for photoelectrons in XPS provided by the avidin layer (there is little nonspecific adsorption of avidin on unexposed PEG as shown by the nitrogen signal in Figure 3F). The oxygen signal intensities for unexposed and exposed (30 sec and 150 sec) PEG samples are similar because the avidin layer somewhat overshadows the small oxygen signal differences observed in Figure 3B. However, since more avidin molecules were found to attach on the exposed (600 sec) sample, its oxygen signal is distinctly different from the latter samples.

**Figure 4:**
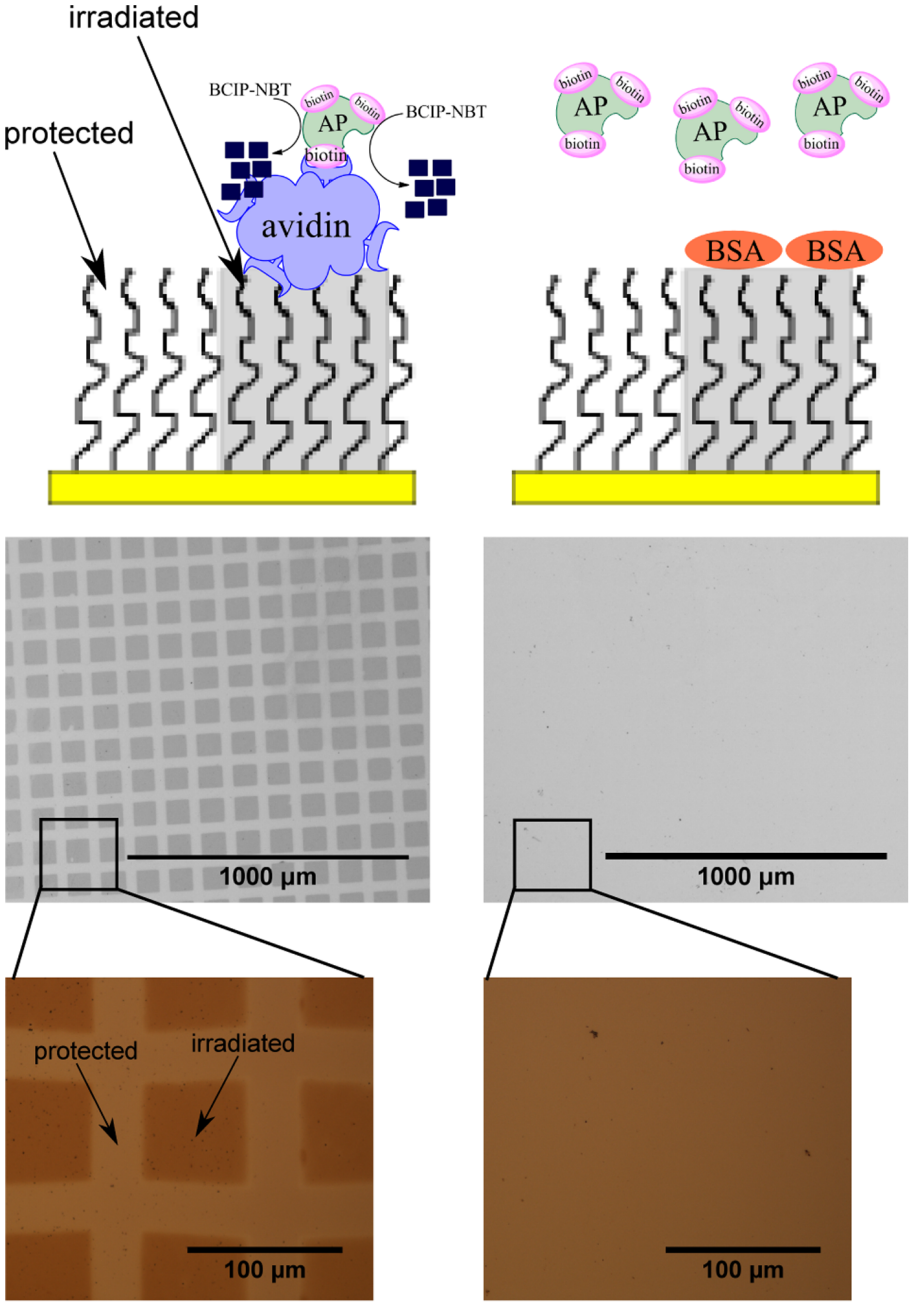
Formation of diformazan precipitates on protein-PEG patterns (35 µm mesh). Top row images show a schematic diagram (not to scale) of helium beam- patterned PEG incubated with avidin (A) or BSA (D) followed by addition of biotinylated alkaline phosphatase (AP) and substrate BCIP-NBT. Optical microscopic images below the diagram show the protected (light) and irradiated (dark) regions which display the specific patterned capture of biotinylated AP enzymes for avidin-incubated surface (B) and no pattern for BSA-incubated surface (E). Panels C and F show higher magnifications of images in B and E, respectively.

**Figure 5:**
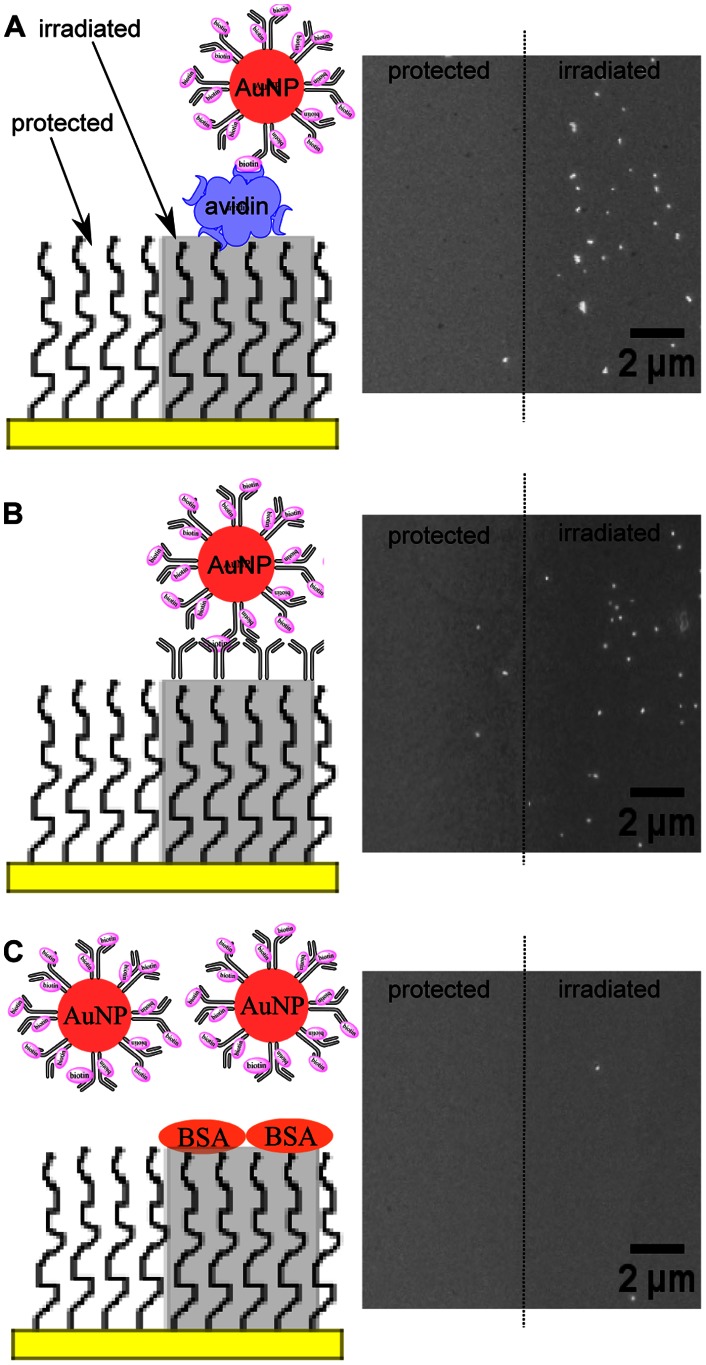
Gold nanoparticle probes on protein-PEG patterns (35 µm mesh). Images on the left column show the schematic diagram (not to scale) of helium beam-patterned PEG incubated with avidin (A), polyclonal anti-rabbit antibodies (B), or BSA (C), followed by the addition of 100 nm gold nanoparticles conjugated with biotinylated rabbit antibodies. Electron microscope images on the right column show the protected and irradiated PEG regions after incubation with gold nanoparticle probes.

Among the helium beam doses tested, we chose to use the 150 second exposure time for further work as a balance between protein attachment effectiveness and processing throughput. Patterning of proteins using a 35 µm mesh is presented in Figures 4 and 5, wherein attachment of proteins on beam-exposed regions is indicated by enzymatic formation of localized diformazan precipitates and also by binding of gold nanoparticle probes. As shown in Figure 4, the helium beam was able to pattern avidin over a large surface area (25 mm^2^, with 2.3 mm^2^ shown in Figure 4). The darkened regions indicate the formation of diformazan precipitates by captured biotinylated alkaline phosphatase via dephosphorylation and reduction of the substrate BCIP-NBT. These regions signify the successful attachment of avidin in active form on beam-exposed PEG. Similar results were obtained using 100 nm gold nanoparticles, as shown in Figure 5A. In Figure 5B, where the surface was incubated with polyclonal anti-rabbit antibodies after beam exposure, gold nanoparticle probes conjugated with biotinylated rabbit antibodies were shown to bind to the irradiated PEG regions. BSA control surfaces, by contrast, showed little to no formation of diformazan precipitate (Figure 4) and bound very few gold nanoparticles (Figure 5C).

**Figure 6:**
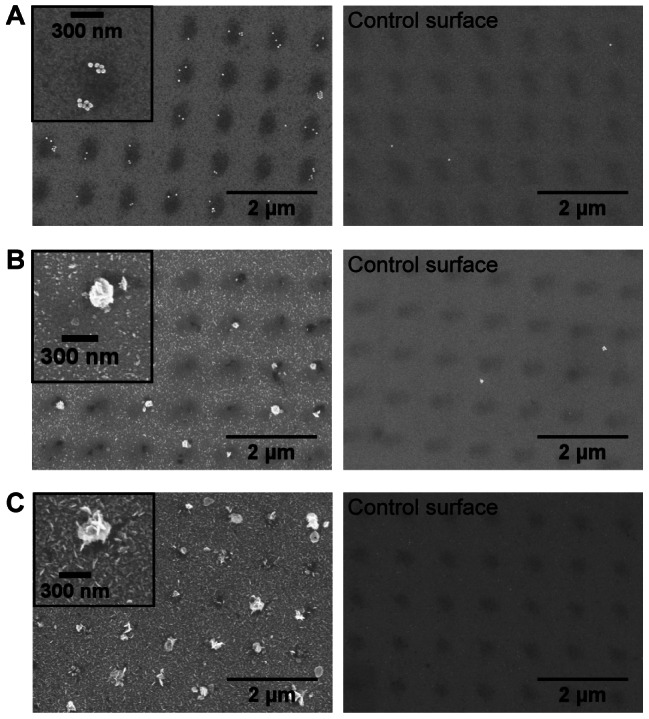
Gold nanoparticle probes and silver nanoparticle deposition on protein-PEG patterns (300 nm mask). Electron microscope images on the left column (with zoom-in pictures as insets) correspond to helium beam-patterned PEG incubated with polyclonal anti-mouse antibodies (A), streptavidin-polyHRP conjugates (B), or biotinylated lysozyme (C), while images on the right display beam-patterned PEG surfaces incubated with PBS buffer (no proteins); all samples were then passivated with BSA. Patterns were visualized by binding of 40 nm gold nanoparticles conjugated with D1.3 mouse antibodies (A), or silver staining through HRP conjugates (B). Captured biotinylated lysozyme (C) was detected by addition of streptavidin-polyHRP conjugates and silver staining.

To demonstrate that the helium beam could create high-resolution nanoscale protein patterns on PEG surfaces, a smaller mask with 300 nm openings at 1 µm spacing was used. As shown in Figure 6A, gold nanoparticles conjugated with monoclonal mouse antibodies were observed to bind to the 300 nm irradiated PEG regions incubated with polyclonal anti-mouse antibodies, while very few bound nanoparticles were seen on a BSA control surface. Similar results were obtained by silver staining, as shown in Figures 6B and 6C. Silver particles of approximately 300 nm diameter were formed on the beam-exposed areas, both on surfaces incubated with streptavidin-HRP, then silver stained with EnzMet^TM^ solution (Figure 6B) and on surfaces incubated with biotinylated lysozyme, followed by streptavidin-HRP conjugate and silver staining (Figure 6C). These results showed that helium beam exposure through stencil masks can be used to activate PEG and create protein patterns on the order of hundreds of nanometers. Corresponding negative controls showed very few bound gold nanoparticles and little to no formation of silver nanoparticles, as BSA was able effectively to passivate the beam-exposed regions.

## Summary

We have demonstrated that helium beam exposure through a stencil mask in proximity to the substrate can be used for the massively-parallel creation of micro- and nano-scale protein patterns on PEG-grafted surfaces. Proteins captured on irradiated PEG regions were shown to retain their functionality, as the patterned avidin could bind biotinylated molecules and patterned HRP conjugates were able to produce silver nanoparticles. Protein attachment on helium beam-exposed PEG may be due to hydrophobic interactions, electrostatic interactions, formation of reactive oxidized products, or a combination of these mechanisms. Further studies will be needed to establish the governing mechanism(s) and also to maximize processing throughput.
